# Supplementary material for: Netcombin: An algorithm for constructing optimal phylogenetic network from rooted triplets
Source: PLoS One. 2020 Sep 18;15(9):e0227842. doi: 10.1371/journal.pone.0227842 (PMC7500971; doi:10.1371/journal.pone.0227842)
Supplement: S4 File — (ZIP) [file pone.0227842.s004.zip › SequenceData-Triplet/sequenceData-10-20-30-0-Triplet/length 1000, numseq 40, rate 10^-7/5 seq.pdf]

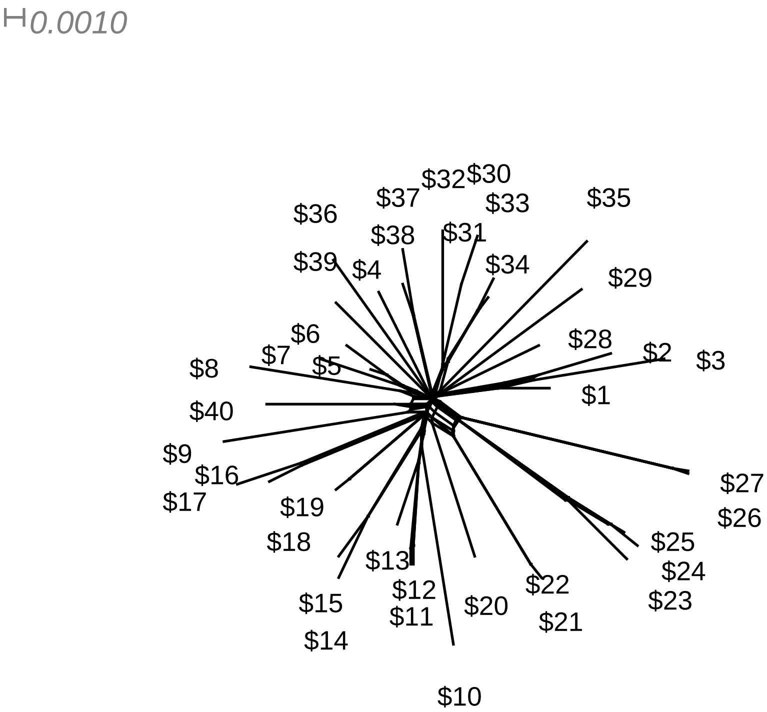
0.0010
$1
$2
$3
$4
$5
$6
$7
$8
$9
$10
$11
$12
$13
$14
$15
$16
$17
$18
$19
$20
$21
$22
$23
$24
$25
$26
$27
$28
$29
$30
$31
$32
$33
$34
$35
$36
$37
$38
$39
$40
